# Right-sided weakness in a Rwandan patient with untreated Tetralogy of Fallot

**DOI:** 10.1186/s12245-023-00494-0

**Published:** 2023-03-14

**Authors:** Sylvain Tshilombo, Romeo Bilugan, Amanda Feeney, Jonathan Im, Heather M. Kuntz, Kavita Gandhi, Besh Barcega, Jean Felix Babane, Vincent Ndebwanimana, Mindi Guptill

**Affiliations:** 1 Department of Anesthesia, Emergency Medicine and Critical Care, University of Rwanda, Kigali, Rwanda; 2 Department of Emergency Medicine, Loma Linda University School of Medicine, 11234 Anderson Street, Loma Linda, CA 92354 USA; 3 Department of Emergency Medicine, University of California, 533 Parnassus Ave, U-575, San Francisco, CA 94143 USA; 4 Department of Emergency Medicine, University Teaching Hospital of Kigali, Kigali, Rwanda

**Keywords:** Tetralogy of Fallot, Pediatrics, Congenital heart disease, Rwanda

## Abstract

**Background:**

Tetralogy of Fallot (TOF) is the most common cyanotic congenital heart disease encountered in pediatrics with surgical repair being the definitive treatment. Long-term survival after surgical repair has improved; however, reported mortality rates in untreated TOF are significant. Associated complications include neurological sequelae such as brain abscess and stroke. In countries without early intervention for congenital heart disease (including TOF), delayed presentations and complications require recognition by healthcare workers.

**Case presentation:**

A 22-year-old male with a history of untreated TOF presented to Rwanda’s tertiary university hospital, University Teaching Hospital of Kigali, with acute right-sided hemiparesis. Diagnostic imaging identified a left-sided brain lesion consistent with brain abscess and cardiac mass, concerning endocardial vegetation. He was managed with intravenous antibiotics but subsequently died due to complications of septicemia.

**Discussion:**

In countries where surgical repair of TOF is not available, early recognition and medical management are key in temporizing the development of devastating sequelae. Describing the prevalence of CHD in Rwanda is urgent, requiring further research by which effective prevention and treatment strategies can be developed.

## Background

Tetralogy of Fallot (TOF) is the most common cyanotic congenital heart disease (CHD) encountered in pediatrics. Research done in high-income countries report TOF is present in approximately 1 in 3500 births and accounts for 7 to 10% of all congenital cardiac malformations. The etiology is multifactorial with up to 25% of cases having chromosomal anomalies (trisomies 13, 18, 21 and microdeletions of chromosome 22) with a familial recurrence rate of 3% [1]. Reparative surgery is the definitive treatment commonly done during the first 6 months to 1 year of life [2]. Screening and surgical advances have allowed for improved long-term survival after TOF repair (85%); however, untreated TOF is associated with increased mortality with only 24% of affected cases surviving past the age of 10 years. Life expectancy for TOF is less than 3% at 40 years if surgically untreated. The major causes of death in untreated TOF include hypoxic spells (62%), strokes (17%), and brain abscesses (13%) [3, 4]

In untreated TOF, each patient’s course is variable and depends on the severity of the right ventricular outflow tract obstruction (RVOT). Increased resistance in the pulmonary circuit caused by the RVOT eventually leads to right ventricular hypertrophy, right ventricular failure, and eventually biventricular failure [3]. Heart failure results in modified cardiac hemodynamics with turbulent flow [5, 6]. Endocardial vegetation formation may happen with turbulent flow leading to endocarditis and thromboembolic events. Infective endocarditis is associated with structurally abnormal hearts and can cause multiple neurologic complications including ischemic stroke, intracerebral hemorrhage, infectious intracranial aneurysms, meningitis, and brain abscess [7, 8].

Brain abscesses are multifactorial in the patient with CHD. Brain abscesses present in 1–7% of patients with infective endocarditis (IE), but the prevalence in patients with IE and untreated TOF is not known. In patients with CHD of all kinds, 5–18.7% of patients have brain abscess because of chronic hypoxia leading to polycythemia, hyperviscosity, poor host immunity and bypass of lung phagocytes [9, 10].

We present the case of a 22-year-old Rwandan male with untreated TOF who presented to the emergency center (EC) of Rwanda’s tertiary university teaching hospital, University Teaching Hospital of Kigali (CHUK). Consent was obtained from the patient prior to his death for the publication of this case report.

## Case presentation

A 22-year-old male who was a local university student presented to the EC of CHUK with a 3-day history of right-sided facial droop and hemiparesis. The patient reported a medical history of untreated TOF; however, the age and circumstances of his initial diagnosis are not known. His symptoms began gradually shortly after awakening 3 days prior and were progressively worsening. He denied precipitating events (e.g., trauma, intoxication, ingestion of unknown substances, or procedures). He also denied concomitant symptoms including confusion, fever, weight loss, headache, chest pain, palpitations, seizure, shortness of breath, or sensory deficits.

Physical examination revealed that the patient was alert, oriented, and in no apparent distress. He had an obvious right-sided facial droop with forehead sparing and decreased strength of both right upper and right lower extremities. Pt noted to have SpO2 of 65% on room air with a heart rate of 68 bpm, blood pressure of 113/58, and temperature of 36.1 °C. Clubbing was noted of the patient’s fingers and toes. Upon auscultation, he had holosystolic cardiac murmur with clear breath sounds. No meningismus was present.

**Fig. 1:**
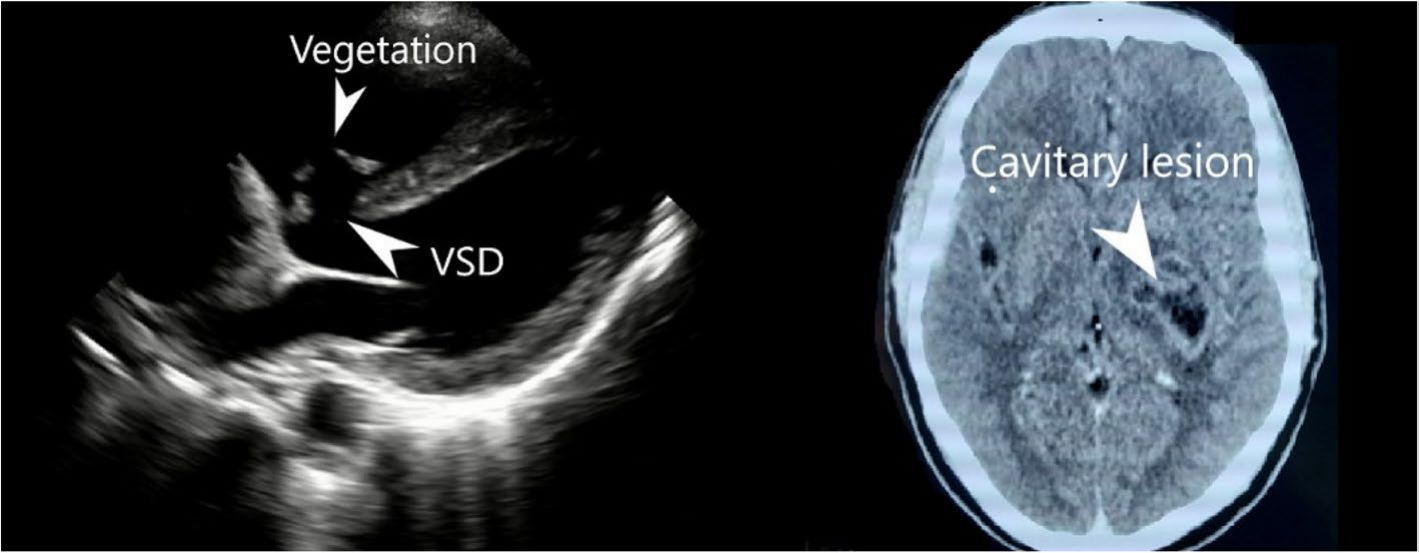
Cardiac POCUS revealing valvular vegetation (left) and brain CT with findings consistent with brain abscess (right)

The patient was evaluated with a non-contrast head computerized tomography (CT) which was remarkable for brain abscess (Fig. 1). Point-of-care ultrasound (POCUS) was performed and revealed a prominent, untreated VSD with a hyper-echoic mass noted in the region where the tricuspid valve abuts the septal defect (Fig. 1). He was started on broad-spectrum intravenous antibiotics for suspected brain abscess and valvular vegetation with concern for infective endocarditis. A CT head with contrast was subsequently obtained and revealed a left-sided irregular focal mass measuring 40 × 28 mm with central necrosis and ring pattern of contrast enhancement.

Laboratory analysis was unremarkable except for a potassium of 6.9 and platelet count of 81. HIV, malaria, and blood cultures were noted to be negative. After stabilization in the EC, he was admitted to internal medicine for continued treatment of hyperkalemia, continued IV antibiotics, and consultation by neurosurgery. His inpatient records did not describe his medical course in detail, but we do know he was reported to have died of septicemia 3 weeks after admission.

## Discussion

The prevalence of CHD, and more specifically of TOF in Rwanda, is not known. CHD in Rwanda is commonly identified after birth based on clinical findings including cyanosis, respiratory distress, diaphoresis with feeding, hepatomegaly, and failure to thrive. The only study on CHD in Rwanda studied children with genetic disorders and found the most common cardiac congenital lesions to be ventricular septal defect, atrioventricular septal defect, and patent ductus arteriosus. In those children with genetic diseases, four cases of TOF were identified: two patients had trisomy 21, one had trisomy 18, and the other had DiGeorge syndrome [11]. Surgical correction of cardiac anomalies in Rwanda has primarily been possible through humanitarian surgical outreach with the average patient age of 25 years [12]. There is no published data specifically about TOF repair in Rwanda.

In low- and middle-income countries (LMICs), CHD is a major cause of infant mortality. The morbidity and mortality of CHD are underestimated in Africa because of poor survivability. CHD management is limited in Africa due to an insufficient technical platform and lack of specialized human resources as patients require a multidisciplinary team including pediatricians, cardiothoracic surgeons, and cardiologists [13]. It is likely that only a small and insignificant portion of the population in LMICs can afford the cost of diagnosis, medical treatment, and/or surgical correction of CHD. The situation is more dire for those living in rural areas where access to basic healthcare is already a serious issue [13, 14].

In situations where children living in LMICs do not have congenital cardiac anomaly screening or are lost to follow-up after diagnosis, TOF may not be surgically corrected. A patient with untreated TOF may present to an EC with neurologic complications. For the patient in this case, the timely use of POCUS identified a valve vegetation; the likely source of the patient’s brain abscess causing right-sided weakness. The importance of a full investigation into possible complications related to untreated CHD cannot be overstated and will aid to target therapy in these patients who are medically complex.

## Conclusion

Recognizing the variety of complex sequela in untreated TOF patients can aid in the EC evaluation and management. Further research on CHD in Rwanda is urgent so that effective prevention and multidisciplinary treatment strategies can be developed.

## Data Availability

Data sharing is not applicable to this article as no datasets were generated or analyzed during the current study.

## Abbreviations

TOF: Tetralogy of Fallot
CHD: Congenital heart disease
EC: Emergency centers
RVOT: Right ventricular outflow tract
LMICs: Low- and middle-income countries
POCUS: Point-of-care ultrasound

## References

[1] Villafañe J, Feinstein JA, Jenkins KJ (2013). Hot topics in tetralogy of Fallot. J Am Coll Cardiol..

[2] Al Habib JF, Jacobs JP, Mavroudis C (2010). Contemporary patterns of management of tetralogy of Fallot: data from the Society of Thoracic Surgeons Database. Ann Thorac Surg..

[3] Bertranou EG, Blackstone EH, Hazelrig JB, Turner ME, Kirklin JW (1978). Life expectancy without surgery in tetralogy of Fallot. Am J Cardiol..

[4] Hokanson JS, Moller JH (1999). Adults with tetralogy of Fallot: long term follow-up. Cardiol Rev..

[5] Nagao GI, Daoud GI, McAdams AJ, Schwartz DC, Kaplan S (1967). Cardiovascular anomalies associated with tetralogy of Fallot. Am J Cardiol..

[6] Stanescu CM, Branidou K (2008). A case of 75-year-old survivor of unrepaired tetralogy of Fallot and quadricuspid aortic valve. Eur J Echocardiogr..

[7] Dobrocky T, Klink T, Weisstanner C, Heverhagen J, Christe A (2014). Imaging findings in uncorrected tetralogy of Fallot and pulmonary atresia with major aortopulmonary collateral arteries and septic embolism. Acta Radiol Short Rep..

[8] Morris NA, Matiello M, Lyons JL, Samuels MA (2014). Neurologic complications in infective endocarditis: identification, management, and impact on cardiac surgery. Neurohospitalist..

[9] Aftab S, Usman A, Sultan T (2015). Frequency of cerebrovascular accidents and brain abscess in children with tetralogy of Fallot. Dr. Pak J Neurol Sci.

[10] Lakhani M, Memon RS, Khan F (2020). Brain abscess: a rare complication in a child with tetralogy of Fallot. IDCases..

[11] Teteli R, Uwineza A, Butera Y (2014). Pattern of congenital heart diseases in Rwandan children with genetic defects. Pan Afr Med J..

[12] Swain JD, Sinnott C, Breakey S (2018). Ten-year clinical experience of humanitarian cardiothoracic surgery in Rwanda: building a platform for ultimate sustainability in a resource-limited setting. J Thorac Cardiovasc Surg..

[13] Tankeu AT, Bigna JJ, Nansseu JR (2017). Prevalence and patterns of congenital heart diseases in Africa: a systematic review and meta-analysis protocol. BMJ Open..

[14] Tantchou Tchoumi JC, Butera G, Giamberti A, Ambassa JC, Sadeu JC (2011). Occurrence and pattern of congenital heart diseases in a rural area of sub-Saharan Africa. Cardiovasc J Afr..

